# Performance of non-binary athletes in mass-participation running events

**DOI:** 10.1136/bmjsem-2023-001662

**Published:** 2023-12-20

**Authors:** John Armstrong, Alice Sullivan, George M Perry

**Affiliations:** 1 Mathematics, King's College London, London, UK; 2 University College London, London, UK; 3 Independent Researcher, Houston, Texas, USA

**Keywords:** Women, Running, Gender

## Abstract

**Objectives:**

To test the hypothesis that, controlling for age, natal-sex differences in running performance are lower among non-binary athletes than in the rest of the population. To test the hypothesis that natal-male non-binary athletes outperform natal-female non-binary athletes.

**Methods:**

A secondary analysis of 166 race times achieved by non-binary athletes within a data set of 85 173 race times derived from races with a non-binary category in the New York Road Runners database. The natal sex of non-binary athletes was modelled probabilistically using US Social Security Administration data when it could not be derived from previous races. Race times were used as the outcome variable in linear models with explanatory variables derived from natal sex, gender identity, age and the event being raced. Statistical significance was estimated using Monte Carlo methods as the model was not Gaussian.

**Results:**

There was no evidence that controlling for age, natal-sex differences in running performance are lower among non-binary athletes. Natal-male non-binary athletes outperform natal-female non-binary athletes at a confidence level of p=0.1%.

**Conclusions:**

Both natal sex and gender identity may be useful explanatory variables for the performance of athletes in mass-participation races. It is, therefore, valuable to include both variables in data collection.

**WHAT IS ALREADY KNOWN ON THIS TOPIC**

- Outside of purely biological outcomes and criminology, little empirical work has been done to test the theory that gender identity is more important than natal sex as a cause of gender disparities in outcomes.

**WHAT THIS STUDY ADDS**

- In mass-participation running, identifying as non-binary does not reduce gender disparities. This provides evidence against the theory that an individual’s gender-identity plays a significant role in these disparities in addition to their natal sex.

**HOW THIS STUDY MIGHT AFFECT RESEARCH, PRACTICE OR POLICY**

- The study highlights the importance of collecting data on both natal sex and gender identity.

## Introduction

Policy debates regarding the relative importance of sex and gender identity have taken place across a wide range of domains, including sports. The literature addresses questions of fairness and participation within sports, particularly relating to the female sports category.^1–5^ In contrast, the current paper seeks to empirically test the respective predictive power of gender identity and natal sex on running performance using the non-binary category. In recent years, several running events have introduced a non-binary category alongside the male and female categories on the grounds that people who identify as non-binary ‘cannot compete authentically within the existing system’.^6^ However, this paper is not intended to study the merits of a non-binary category. Our interest in the category arises because it gives us a data set that can be used to test gender-identity theory.

It is contested whether sex is ‘binary’,^7 8^ a ‘multidimensional biological construct’,^9^ or whether ‘the ‘naming’ of sex is an act of domination and compulsion, an institutionalised performative that both creates and legislates social reality by requiring the discursive/perceptual construction of bodies in accord with principles of sexual difference’.^10^ For this paper, it is not necessary to take sides in this debate. We will use ‘sex’ to refer to sex registered at birth, and we use the terms natal male and natal female to refer to sex registered at birth.

According to gender-identity theory, everybody has an innate gender identity, defined by UK LGBTQ+ (lesbian, gay, bi, trans, queer, questioning and ace) Charity Stonewall as ‘A person’s innate sense of their own gender, whether male, female or something else … which may or may not correspond to the sex assigned at birth.’.^11^ Advocates of gender-identity theory argue that gender identity is typically more important than sex in determining outcomes, which are socially influenced, an idea sometimes expressed as ‘trans women are women’.^12^ For example, according to Safer,^13^ ‘for general categorisation by sex, ‘brain sex’ or gender identity would be the default characteristic’. Such viewpoints have been widely adopted, leading to some statistical bodies advising that data on sex should not be collected.^14–18^ Yet the question of the respective importance of sex and gender identity on relevant outcomes and the interaction between the two is an empirical one that cannot be answered without data on both gender identity and sex. The existing literature is limited and, in both sports and criminology, typically relates to the effects of medical transition rather than identity per se.^19–21^

Running counter to gender-identity theory is the gender-critical belief ‘that biological sex is real, important, immutable and not to be conflated with gender identity’^22^ and that sex often has important effects that cannot be explained purely by gender identity. We acknowledge that not everyone who holds these views would choose to label themselves as gender critical.

We will consider sporting performance as a test case for these theories. In mass-participation events, social factors, such as levels of training, personal motivation, personal expectation and natural competitiveness, are likely to impact sporting performance significantly. Even for elite athletes who do not lack motivation and competitiveness, there are likely to be gendered differences caused by issues such as family commitments, the availability of sponsorship and levels of access to coaching and individualised sports science. Such issues have been studied extensively in the literature on gender and sport.^23 24^ The potential importance of social determinants in sports is also highlighted in.^9^ Suppose gender identity influences how one performs social roles.^10^ Since the sports literature indicates that gendered roles influence sports performance, in that case, one should expect that gender identity may be associated with sports performance.

We will study the application of gender-identity theory to road-racing data gathered by New York Road Runners (NYRR), whose races in recent years have included three categories: male, female and non-binary. Their non-binary category is a simple matter of self-identification as either identifying as non-binary or not, though non-binary identities are varied and complex.^25 26^ One definition of non-binary is ‘an adjective describing a person who does not identify exclusively as a man or a woman. Non-binary people may identify as being both a man and a woman, somewhere in between, or as falling completely outside these categories. While many also identify as transgender, not all non-binary people do. Non-binary can also be used as an umbrella term encompassing identities such as agender, bigender, genderqueer or gender-fluid.’.^27^ The qualitative literature on non-binary identities and sports suggests that people who identify as non-binary face barriers to participation but typically does not interrogate how these experiences vary by sex.^28–31^

## Methods

We will test hypotheses derived from gender-identity theory and gender-critical theory by examining how similar the performance of athletes in the non-binary category is to that of other athletes of the same sex.

Our first hypothesis derives from gender-identity theory.

### Hypothesis 1

Controlling for age, sex differences in non-binary athletes’ race times will be smaller than the sex differences in race times observed for other athletes.

Our second hypothesis derives from the gender-critical view.

### Hypothesis 2

Controlling for age, natal-female non-binary athletes will tend to have slower race times than natal-male non-binary athletes.

Note that hypothesis 1 is derived from, but not equivalent to, gender-identity theory. Similarly, hypothesis 2 is derived from, but not equivalent to, gender-critical theory. Thus, these hypotheses are natural tests to perform from the point of view of falsifying each of the theories. Note also that these hypotheses are not mutually exclusive: it is possible that both sex and gender-identity have significant effects.

Unfortunately, the sex of athletes who identify as non-binary is not recorded in our data set. To overcome this obstacle, we use the novel technique of modelling the likely sex of athletes based on their given names. We do not claim that one can perfectly predict sex based on an athlete’s given name. Instead, we develop a probability model for sex. Our technique is similar to the accepted statistical practice of imputing missing values in a data set.^32^ We will show through cross-validation that our probability model can be used to predict the natal sex of non-binary athletes. In online supplemental appendix C, we repeat our analysis using a probability model that includes additional uncertainty from causes such as athletes changing their names. This increases the estimate for the size of sex differences among non-binary athletes and, if one assumes sufficient uncertainty would suggest a statistically significant increase in sex differentials, the opposite effect to that predicted by gender-identity theory. This should be viewed simply as showing that our model is not skewed in favour of gender-critical theory.

10.1136/bmjsem-2023-001662.supp1 Supplementary data

We also performed exploratory analyses of other potential associations with gender identity, discussed below.

### Data

We used race data from the NYRR database,^33^ selecting races that featured non-binary athletes giving 21 races listed online in online supplemental appendix A. This data set was selected as the largest available consistently formatted data on non-binary athletes. Before embarking on the study, we performed a power analysis that indicated the data set would be sufficient to detect if the times of natal-male and natal-female athletes were equal in the non-binary category (based on NYRR marathon data, we estimated that the difference in the mean race times was 24.6 min with an SD of 58 min, we estimated that a total sample size of 138 was required to achieve 80% power at 95% CI for a one-tailed t-test). Where possible, the sexes of non-binary athletes with a given name were identified using the Athlinks website.^34^ The frequencies of different baby names in each year in the USA were obtained from the US Social Security Administration baby name database.^35^

### Creation of cross-race data set

Because we were using data from multiple races, the same athlete had often competed in multiple races. We assumed two athletes of different races with the same name were the same athlete.

We wished to create a data set, which contained only one record for each athlete. We decided to base our decision on which result to choose for a given athlete by choosing the race most closely correlated to the marathon once outliers had been discarded. Our assumption that athletes with the same name were the same athlete may have led us to unnecessarily discard some records reducing the sample size and statistical power slightly.

**Figure 1:**
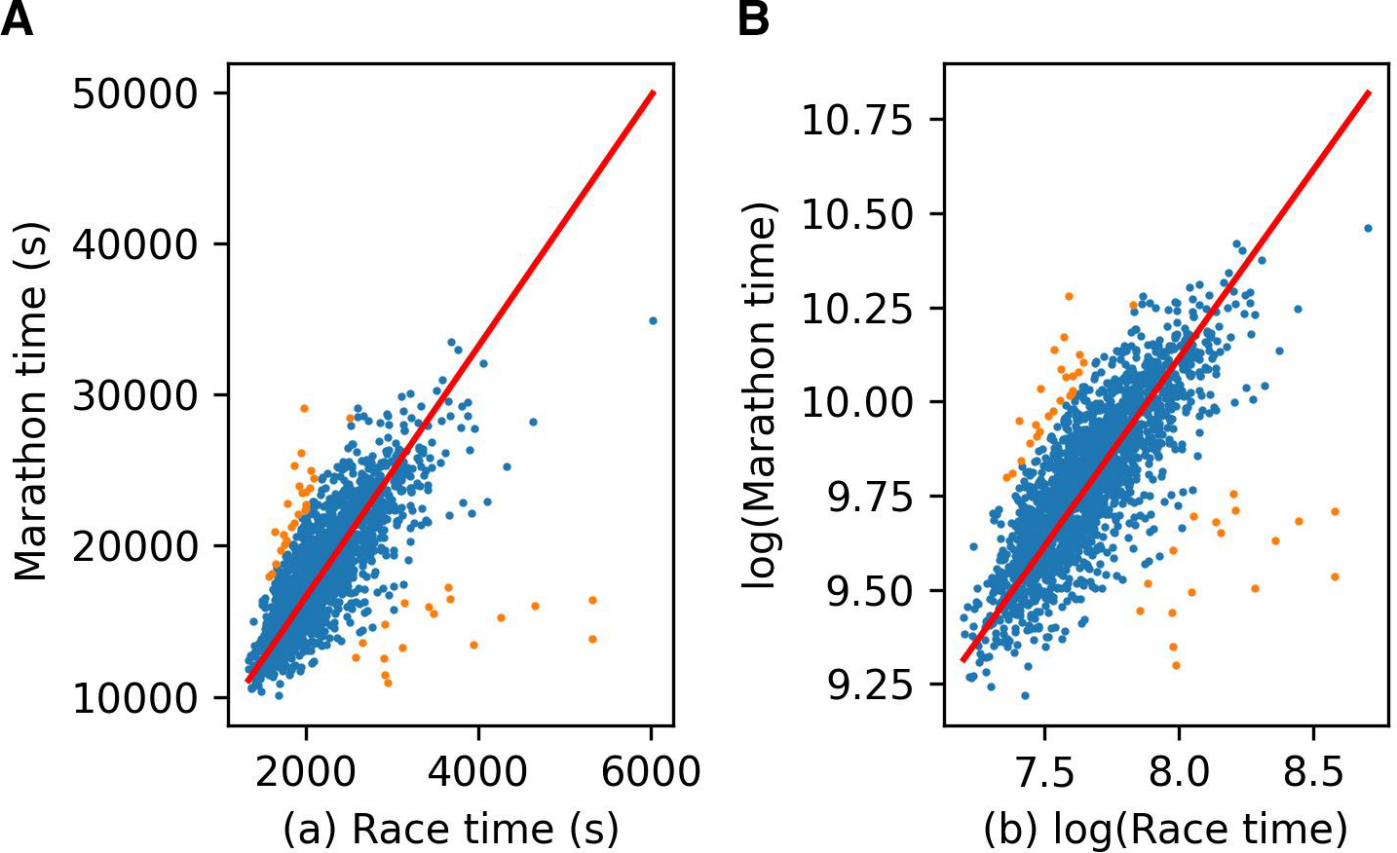
(A) Race time in the ‘Run as One 4M’ plotted against marathon time for athletes who completed both events. The slope of the red line was then estimated using points in blue. The 2% of orange points were treated as outliers, which were ignored in this computation. The red line gives the predicted marathon times for the race. (B) The same data were plotted but using a logarithmic scale.

In more detail, for each race, we identified the athletes who had also run the New York Marathon. We then fit a linear model with no intercept, which allowed us to estimate an athlete’s marathon time based on their race time. We then fit a second linear model to the 98% of times having the lowest values for the ratio of the residual to their time. These second linear models allowed us to predict any runner’s marathon time based on their time in the race; we will just call this the predicted marathon time. See figure 1 A for an example. We will call the correlation coefficient of this time the marathon correlation of the race.

We selected the race they had run with the highest marathon correlation for each athlete, allowing us to obtain a data set with one entry per athlete. The race selected was always the marathon for athletes who had competed in the marathon. We then discarded extreme outlying data points where the predicted marathon time was greater than 11 hours, the cut-off time for the New York Marathon. This gave us our cross-race data set.

Note that while the orange points shown in figure 1A were discarded to estimate marathon times, those records were still eligible for inclusion in the cross-race data set. Fewer than 0.1% of records were discarded as outliers in creating the cross-race data set. See table 1 for a summary of the number of data points used to create our cross-race data set.

**Table 1:** Summary of the number of records used in the creation of the cross-race data set

Total no of race results in full data set	186 782
No of outliers discarded	159
No of unique rows in cross-race data set	85 173

### Modelling sex

NYRR does not record the sex of non-binary athletes. We addressed this by developing a probability model for the sex of each competitor. We did this by computing a variable prob_male, which contained our estimate of the probability that an athlete is natal-male.

For athletes who stated they were male or female, we set prob_male to be either 0 or 1. For non-binary athletes, we checked if we could find that athlete’s sex on the Athlinks website. In this case, we set prob_male to be either 0 or 1.

If we could not find the athlete on the Athlinks website, we used a database of US baby names to determine the proportion of male babies born in the US with the same first name as the athlete. We used this proportion to determine the value of prob_male. To be precise, we computed the two possible years in which the athlete was born based on their age on race day and the date of the race and used the proportion of babies born in those 2 years.

Runners where none of these methods provided any information about their sex were excluded from the analysis.

A summary of how each non-binary athlete’s sex was identified is given in table 2.

**Table 2:** Summary of how we estimated the probability that a non-binary athlete was a natal-male

	No
Sex found using Athlinks	92
Probability estimated using baby name database	53
No probability assigned	21
Total	166

We cross-validated the data from Athlinks with the probability model based on baby names. We looked at the athletes where the probability of them being a natal male was less than 0.05 or greater than 0.95 according to the baby-name database and assumed for our cross-validation that these athletes were either natal females or natal males. We found that in 77 of 78 cases where both methods allowed us to compute the athlete’s sex, both methods gave the same answer.

The final cross-race data set contained records, each representing a different athlete. Each record consisted of a field event indicating which race was being run, a field gender_id indicating whether the athlete had registered as either male, female or non-binary), a field prob_male, their race time in seconds and their age.

### Linear modelling

**Figure 2:**
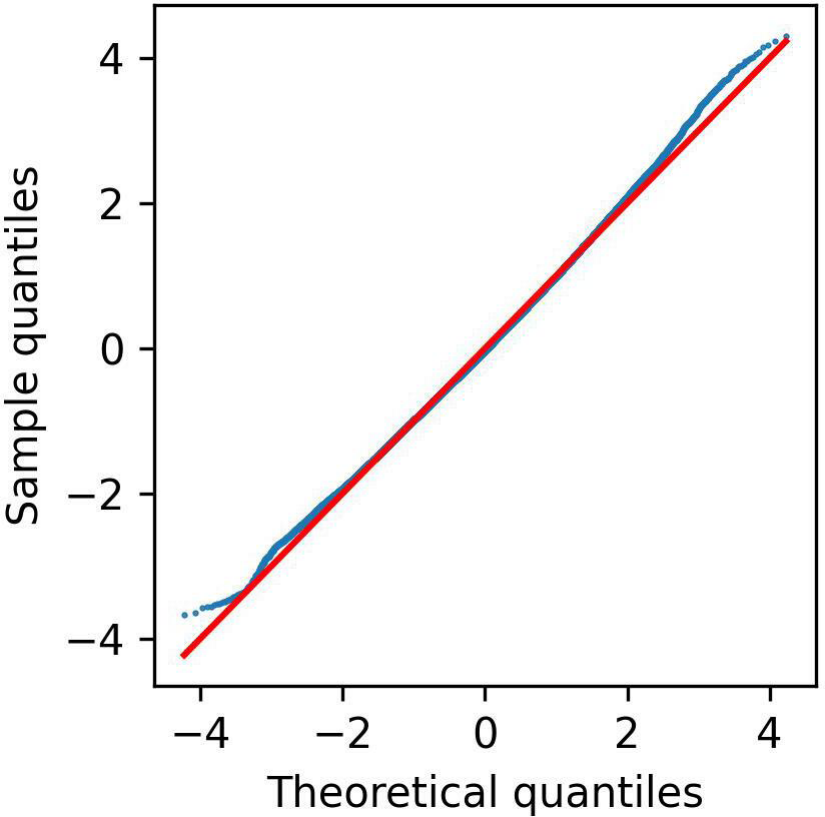
Normal Q-Q plot of the residuals for the model given by equation 4 for male and female athletes.

We modelled the logarithm of times rather than the times themselves. It is visually clear that the times plotted in figure 1A do not follow a multivariate normal distribution. Transforming figure 1A using a log-log plot results in the data shown in figure 1B, which is approximately normal. We see in figure 2 that modelling the logarithm of the race times rather than the race times leads to the residuals in our final model having an approximately normal distribution. It also meant we could control for the different lengths and difficulty of races simply by including an event variable in our models.

We assumed there was an unobserved variable called natal_sex, which took one of two values, ‘natal male’ or ‘natal female’. For each runner, the probability of this variable taking the value male was assumed to be given by the variable prob_male.

In online supplemental appendix B, we describe how to estimate the coefficients of a linear model which contains a binary unobserved variable and where some of the explanatory variables depend on this unobserved variable. The interpretation of the resulting coefficients and the p values are the same as in a conventional linear model.

### Analysis

#### Hypothesis 1

To test the hypothesis that non-binary athletes’ performance is less determined by their sex than other athletes, we created a variable nb_predictor, which took the value 1 for non-binary athletes who are natal males, −1 for non-binary athletes who are natal females and 0 otherwise. We then fit the following linear model:

$log(time)∼event+natal\_sex+age+age^{2}+nb\_predictor$(1)

The coefficients obtained for all our models can be seen in table 3. The coefficients for the intercept and the different running events are all highly significant but have been omitted as they are irrelevant to our hypotheses. We have shown p values for both one-tailed and two-tailed tests. The p values for the one-tailed tests are the ones that we use for our hypothesis testing. The two-tailed tests are the relevant values for our exploratory analyses. We have included a column showing the effect sizes as percentage increases in race times for ease of interpretation.

**Table 3:** Coefficient estimates for each of our linear models

Parameter	Coefficient	Effect size	P value (one tailed)	P value (two tailed)
Model 1: event+natal_sex+(age−40)+(age−40)^2^+nb_predictor				
sex=natal female	0.12221	13.00%***	0.0000	0.0000
(Age−40)	0.00375	0.38 %/y***	0.0000	0.0000
(Age−40)^2^	0.00012	0.012 %/y^2^***	0.0000	0.0000
nb_predictor	−0.00424	−0.42 %	0.4071	0.8079
Model 2: event+gender_id+(age−40)+(age-40)^2^+nb_predictor				
gender_id=’female’	0.12222	13.00%***	0.0000	0.0000
gender_id=’non-binary’	0.09387	9.84%***	0.0000	0.0000
(Age−40)	0.00375	0.376 %/y***	0.0000	0.0000
(Age−40)^2^	0.00012	0.012 %/y^2^***	0.0000	0.0000
nb_predictor	−0.06803	−6.58%***	0.0001	0.0001
Model 3: event+gender_id+(age−40)+(age−40)^2^+is_nbm+is_nbf				
sex=natal female	0.12222	13.00%***	0.0000	0.0000
(Age−40)	0.00375	0.376 %/y***	0.0000	0.0000
(Age−40)^2^	0.00012	0.012 %/y^2^ ***	0.0000	0.0000
is_nbm	0.02584	2.62%	0.1324	0.2681
is_nbf	0.03969	4.05%	0.0580	0.1152
Model 4: event+natal_sex+(age−40)+(age−40)^2^+isNB				
isNB	0.03225	3.278% (.)	0.0262	0.0528
natal_sex=natal female	0.12224	13.002 %/y***	0.0000	0.0000
(Age−40)	0.00375	0.376 %/y^2^***	0.0000	0.0000
(Age−40)^2^	0.00012	0.012% ***	0.0000	0.0000

Coefficients for different events are ignored. The coefficients are given as a percentage increase in marathon time in the ‘effect size’ column for ease of interpretation. The final two columns contain Monte Carlo estimates for the p values of the coefficients estimated using 100 000 samples.

The symbols (.), *, **, *** indicate statistical significance at the 0.10, 0.05, 0.01 and 0.001 levels using a two-tailed test.

We have stated the coefficients of the age terms relative to 40 which is, to a good approximation, the mean age of athletes. Thus, the (age−40) coefficient represents the annual deterioration in performance at this mean age.

We used a quadratic model used for age as the data shows performance peaks at approximately age 25 then deteriorates gradually till 40 and then more sharply. With this quadratic model for age, we found no interaction terms for sex and age. We also analysed the data with a more complex piecewise-linear model age, but this made no material difference to our findings.

If sex differences in race times were reduced among non-binary athletes, the sign of the coefficient for nb_predictor would be positive. However, the sign of the coefficient is negative. Thus, hypothesis 1 is not supported by our data. However, the coefficient is not statistically significant, so we cannot conclude that the sex differences in performance are greater for non-binary athletes.

#### Hypothesis 2

To test hypothesis 2, we fit the following linear model:

$log(time)∼event+gender\_id+age+age^{2}+nb\_predictor$(2)

In this case, the variable nb_predictor was significant at a 0.1% confidence level. Hence, we can reject the null hypothesis, and it appears that the performance of non-binary athletes is affected by their sex.

### Exploratory analyses

To see if there were any other discernible associations with being non-binary, we fit the following linear model:

$log(time)∼event+natal\_sex+age+age^{2}+is\_nbm+is\_nbf$(3)

Where is_nbm and is_nbf indicate if an individual is a natal male and non-binary or a natal female and non-binary, respectively, neither is_nbm nor is_nbf was significant at a 5% confidence level. The coefficients were of a very similar size. Thus, our sample size is not large enough to discern the individual effects of being natal-male and non-binary or being natal-female and non-binary, and there is no evidence of any differential effect of being non-binary between these categories.Our final choice of model is:

$log(time)∼event+natal\_sex+age+is\_nb+age^{2}$(4)

Where is_nb is a variable that indicates whether or not an athlete is non-binary.

One sees from the coefficients that being a natal female, whether or not they are non-binary, is associated with an increase in race times of approximately 13%. Being non-binary may be associated with an increase in race times of approximately 3.3% but as this is on the boundary of the 5% significance level using a two-tailed test one would wish to examine a larger sample to explore this further. The residual SD in our model for the logarithm of race times is 0.20, which corresponds to a 22% change in race times. Thus, for all athletes, the differences within sex categories are larger than the differences between sex categories.

A q-q plot for the residuals of the model given by equation 4 for the athletes whose sex is known is shown in figure 2. This shows a good degree of normality for the residuals, validating this assumption of our hypothesis testing.

## Discussion

Our results illustrate the value of data on sex and gender identity.

The differential between natal male and natal female performances is better explained by differences in sex than differences in gender identity, as this differential persists for our non-binary cohort. This provides evidence against the theory that an individual’s gender-identity plays a significant role in these disparities in addition to their sex.

Our exploratory analysis indicates that non-binary athletes may have slower race times than other athletes once one controls for sex and age, but one would wish to confirm this with a larger data set as this is on the boundary of statistical significance. Data gathered on gender non-conforming college students by the American College Health Association^36^ suggest that gender non-conforming students are less likely to meet exercise recommendations, have increased rates of obesity and have higher rates of physical and mental health issues; these factors affect levels of fitness and training status. A complex range of factors associated with non-binary status could account for any association with slower race times. We do not wish to suggest causality in either direction.

### Research implications

Any possible differential in the performance of non-binary athletes would be masked if one did not consider sex. This illustrates that if one wishes to understand the needs of gender non-conforming individuals, it is vital to control for sex as it is likely to play a significant role in any analysis.

The prediction arising from gender-identity theory that sex differences in performance will be lower among non-binary athletes in mass-participation running events is not supported by our results. Our study illustrates the importance of controlling for sex when studying gender non-conforming individuals in the context of sports performance. When considering gender-identity theory in any context, one cannot rely purely on theoretical arguments to determine whether gender-identity or sex is the more significant factor.

Given the lack of empirical evidence supporting gender-identity theory, one should not assume by default that gender-identity is a more powerful explanatory variable than sex. Being an objectively measurable binary variable, sex has considerable explanatory advantages over gender identity.

Our study illustrates that gender identity may be significant as a variable even in situations where the data do not support the predictions of gender-identity theory. It would be interesting to see in a larger study whether non-binary athletes do have slower race times controlling for sex and age, if so this would justify our initial assumption that social factors have a significant impact on sporting performance.

In summary, our study shows the value of gathering data on sex and gender-identity. It illustrates how this is particularly necessary if one wishes to understand the experiences of gender non-conforming individuals. A similar view is put forward in Hunter *et al*.^9^

### Limitations

Non-binary runners may have chosen to run as either male or female athletes. Thus, our analysis only applies to those non-binary athletes who chose to run in the non-binary category. The race categories conflated sex categories (male and female) with gender identity categories such as non-binary. Our sample of non-binary athletes is not much larger than needed to detect sex differences, so if gender identity differences exist but are smaller than sex differences our study may not have had sufficient power to detect them. Our analysis has grouped all non-binary identities into a single category, if sufficient data and suitable categories were available, a more nuanced analysis of non-binary identities might reveal effects for specific forms of non-binary identity.

## Data Availability

Data are available on reasonable request.
