# Supplementary material for: Performance of non-binary athletes in mass-participation running events
Source: BMJ Open Sport Exerc Med. 2023 Dec 20;9(4):e001662. doi: 10.1136/bmjsem-2023-001662 (PMC10749040; doi:10.1136/bmjsem-2023-001662)
Supplement: Supplementary data [file bmjsem-2023-001662supp001.pdf]

Appendix A

The races included in our data set were:

Race New York Marathon
NYRR Staten Island Half
New Balance Bronx 10M
RBC Brooklyn Half
NYRR Team Championships 5M
NYRR Grete's Great Gallop 10K
Mastercard New York Mini 10K
NYRR Queens 10K
Fred Lebow Half-Marathon
NYRR Ted Corbitt 15K
Percy Sutton Harlem 5K
NYRR Washington Heights Salsa, Blues, and Shamrocks 5K
Italy Run by Ferrero 4M
Gridiron 4M presented by The FLAG Art Foundation
NYRR Joe Kleinerman 10K
NYRR Manhattan 10K
Front Runners New York LGBT Pride Run 4M
New Balance 5th Avenue Mile
Run as One 4M presented by JPMorgan Chase
Achilles Hope & Possibility 4M; NYRR Al Gordon 4M
NYRR Newport 5K

We excluded the "SHAPE + Health Women's Half Marathon" as it was a women-only event and the "TCS New York City Marathon Training Series 12M" as it was a training event rather than a race.

Appendix B – Linear modelling with a hidden variable

Suppose that from our sample we can compute the values of d_1 exogenous variables in $X^1, X^2, \ldots, X^{d_1}$. Suppose that there are d_2 additional variables $Y^1, Y^2, \ldots, Y^{d_2}$ which we cannot compute directly from our sample because they depend upon a hidden Bernoulli variable J, but we do know that we compute the values $T^1, T^2, \ldots, T^{d_2}$ that they will take if the Bernoulli variable is equal to 0 and we can also compute the values $F^1, F^2, \ldots, F^{d_2}$that they will take if the hidden Bernoulli variable is equal to 0. We also have an exogenous variable p that contains the probability with which the value 1 occurs.

In our applications, J takes the value 1 if the athlete is female and 0 if the athlete is male. The variable Y^1 might then represent the sex of the athlete and may also take the value 1 if the athlete is female and 0 otherwise. In this case $T^1 = 1$ and $F^1 = 0$. The variable Y^2 might be used to represent the interaction between sex and age, so we would set T^1 to equal the variable **age** and F^1 to equal 0.

We would like to model an endogenous variable Z using a linear model in our exogenous variables of the form:

$$Z = \sum_{i=1}^{d_1} \beta_i X^i + \sum_{i=1}^{d_2} \gamma_i Y^i + \varepsilon$$

where the ε are assumed to be independent identically distributed random variables of finite variance and mean 0. We assume these variables are also independent of J. This is the form of model used when we describe our analysis in this paper. When we come to compute P-values, we will further assume that the ε follow a normal distribution.

Since $Y^i = J\,(T^i - F^i) + F^i$ we may rewrite this equation as follows:

$$Z = \sum_{i=1}^{d_1} \beta_i X^i + \sum_{i=1}^{d_2} \gamma_i \big(p(T^i - F^i) + F^i\big) + \varepsilon + \sum_{i=1}^{d_2} \gamma_i \big(J\,(T^i - F^i) - p(T^i - F^i)\big)$$

Equation 1

If we define a new exogenous variable

$$P^i \coloneqq p(T^i - F^i) + F^i$$

and a new hidden variable

$$\tilde{\varepsilon} \coloneqq \varepsilon + \sum_{i=1}^{d_2} \gamma_i \big(J\,(T^i - F^i) - p(T^i - F^i)\big)$$

then our model becomes a more familiar linear model where all terms are known in the sample apart from the single random term $\tilde{\varepsilon}$.

$$Z = \sum_{i=1}^{d_1} \beta_i X^i + \sum_{i=1}^{d_2} \gamma_i P^i + \tilde{\varepsilon}.$$

Equation 2

Since the expectation of J is equal p, we see that expectation of $\tilde{\varepsilon}$ is zero. This means we may use ordinary least squares to estimate the coefficients in this regression. However, the $\tilde{\varepsilon}$ will no longer be identically distributed: instead, they will have variance given by

$$V \coloneqq \operatorname{Var} \tilde{\varepsilon} = \left(\sum_{i=1}^{d_2} \gamma_i (T^i - F^i)^2 \right)^2 p(1-p) + \operatorname{Var} \varepsilon.$$

Equation 3

As a result, the ordinary least squares estimator for Equation 2 will not be an optimal estimator in the sense of the Gauss-Markov theorem. To obtain a better estimation of our coefficients we adopt a two-stage estimation process which we will now describe.

First, we use ordinary least squares to get a first estimate of the γ_i. Next, we estimate $\operatorname{Var} \varepsilon$ using the equation:

$$\text{N Var}\,\varepsilon + \sum_{\alpha=1}^{N}\left(\sum_{i=1}^{d_2}\gamma_i(T^i - F^i)^2\right)^2 p(1-p) += \sum_{\alpha=1}^{N} V_\alpha \approx \frac{N}{N-d_1-d_2}\sum_{\alpha=1}^{N} r_\alpha^2.$$

Equation 4

In this equation, N denotes the sample size, the index α runs over the items in the sample and r_α denotes the residual for item α. This equation is derived from Equation 3 combined with the standard estimate for the total variance of the model. Using Equation 3 we now know the variance of the $\tilde{\varepsilon}$.

The second step of our estimation process is to re-estimate the coefficients of our model using weighted least squares with weights $\frac{1}{V_\alpha}$. By the Gauss-Markov theorem this should approximate the best linear approximator. We can also compute a new estimate for improved estimator for Var ε using a weighted version of Equation 4:

$$\left(\text{Var}\,\varepsilon \sum_{\alpha=1}^{N}\frac{1}{V_\alpha}\right) + \sum_{\alpha=1}^{N}\frac{1}{V_\alpha}\left(\sum_{i=1}^{d_2}\gamma_i(T^i - F^i)^2\right)^2 p(1-p) \approx \frac{N}{N-d_1-d_2}\sum_{\alpha=1}^{N}\frac{1}{V_\alpha}r_\alpha^2$$

To compute the P-values of the coefficients, we used Monte Carlo simulations We then computed the P-values using a 2-tailed test with a Monte Carlo simulation assuming that the ε are normally distributed. In detail, we simulated J and ε on the basis of this assumption, and so could compute Z using Equation 1 for any desired choice of coefficients β and γ. To compute the P-value of a particular coefficient, we performed Monte Carlo simulations under the null hypothesis that the coefficient was instead 0. We then counted the proportion of occasions on which our estimation procedure gave a larger coefficient value than the parameter estimate arising from the data.

Appendix C – Modelling additional uncertainty

We have described in the paper how we computed a variable called **prob_male** which we use to model the probability that an athlete is natal-male based on their name and race category. There is no “correct” model for this probability and other models can be proposed. In particular, since athletes may have changed their name since birth, one might want to add in some additional uncertainty to the model to reflect that. A simple way to do this is to choose a parameter value α which takes values between 0 and 0.5 and to then define a new variable **q** as follows:

$$\mathbf{q} = \begin{cases} \mathbf{prob\_male}, & \text{athlete is not non-binary} \\ \alpha + (1-2\alpha)\mathbf{prob\_male}, & \text{athlete is non-binary.} \end{cases}$$

The variable **q** models the probability that an athlete is male, with some additional uncertainty added over and above that arising from the distribution of given names. If a non-binary athlete has a name which is only used for females, then the **q**-model probability of them being a natal male will be α. If they have a name which is only used for males, then the **q**-model probability of being a natal male will be $1-\alpha$. Hence α is a measure of the uncertainty in the **q**-model on top of the uncertainty arising from their name alone.

If we re-run our analysis using **q** in place of **prob_male**, we can test our hypotheses using this alternative probability model. The results are shown in Table 5 in the case when $\alpha = 0.05$. Given how successful our cross-validation was at predicting the natal sex of non-binary athletes, we feel

this choice of α is probably an over-estimate of the uncertainty arising from issues such as changes of name.

Parameter	Coefficient	Effect size	P value (1 tail)	P value (2 tail)
Model 1: **event** + **natal_sex** + (**Age**-40) + (**Age**-40)^2 + **nbPredictor**				
**natal_sex**='natal_female'	0.12221	13.0 % ***	0.0000	0.0000
(**Age**-40)	0.00375	0.38 %/y ***	0.0000	0.0000
(**Age**-40)^2	0.00012	0.012 %/y^2***	0.0000	0.0000
**nbPredictor**	-0.06957	-6.7 % *	0.0227	0.0466
Model 2: **event** + **gender** + (**Age**-40) + (**Age**-40)^2 + **nbPredictor**				
**gender**='female'	0.12221	13.0 % ***	0.0000	0.0000
**gender**='non-binary'	0.09388	9.84 % ***	0.0000	0.0000
(**Age**-40)	0.00375	0.38 %/y ***	0.0000	0.0000
(**Age**-40)^2	0.00012	0.012 %/y^2***	0.0000	0.0000
**nbPredictor**	-0.13608	-12.723 % ***	0.0001	0.0001
Model 3: **event** + **natal_sex** + (**Age**-40) + (**Age**-40)^2 + **is_nbm** + **is_nbf**				
**natal_sex**='natal female'	0.12221	13.0 % ***	0.0000	0.0000
(**Age** -40)	0.00375	0.38 %/y ***	0.0000	0.0000
(**Age** -40)^2	0.00012	0.012 %/y^2***	0.0000	0.0000
**is_nbm**	-0.04220	-4.13 %	0.1320	0.2617
**is_nbf**	0.10775	11.4 % **	0.0035	0.0075
Model 4: **event** + **natal_sex** + (**Age**-40) + (**Age**-40)^2 + **isNB**				
**isNB**	0.02994	3.0 %	0.0363	0.0727
**natal_sex**='natal female'	0.12227	13.0 % ***	0.0000	0.0000
(**Age** -40)	0.00375	0.376 %/y ***	0.0000	0.0000
(**Age** -40)^2	0.00012	0.012 %/y^2***	0.0000	0.0000

Table 5: Coefficient estimates for each of our linear models using the ***q****-model when α=0.05. The final two columns contain Monte Carlo estimates for the P-values of the coefficients estimated using 100,000 samples. The asterisks indicate statistical significance at the 0.05, 0.01 and 0.001 levels using a 2-tailed test.*

Using the **q**-model with $\alpha = 0.05$ we find that there is a statistically significant widening of the sex gap in athlete's performance for non-binary runners. When we choose an implausibly high value for the uncertainty α this effect is more exaggerated. This property of our model is easily explained: the range of the predictor variable (the probabilities of being male or female) is smaller in the **q**-model than in the original model, but the range of the outcome variables (race times) are unchanged. As a result, one should expect a larger magnitude for the coefficient of **nb_predictor** in the **q**-model and one expects its magnitude to increase as α increases. This shows that our original approach of ignoring the uncertainty arising from athlete's changing may lead us to slightly under-estimate the magnitude of sex differences in running performance among non-binary athletes, but assuming that $\alpha < 0.05$, any bias introduced this way will be a relatively small.
